# Effect of mindfulness meditation on depression during pregnancy: A meta-analysis

**DOI:** 10.3389/fpsyg.2022.963133

**Published:** 2022-09-14

**Authors:** Yuchao Li, Jinghui Chen, Baoxia Chen, Tingting Wang, Zhusheng Wu, Xia Huang, Shanshan Li

**Affiliations:** ^1^Sports Department, University of Electronic Science and Technology of China, Chengdu, China; ^2^School of Physical Education, Sichuan University, Chengdu, China; ^3^Sports Department, Chengdu Normal Primary School Attached Huarun, Chengdu, China

**Keywords:** mindfulness meditation, pregnancy, depression, meta-analysis, randomized controlled trial

## Abstract

**Purpose:**

This study systematically evaluates the effect of mindfulness meditation on depression during pregnancy. We provide evidence-based suggestions for preventing and reducing depression during pregnancy by exploring the most effective intervention mode, cycle, and frequency of mindfulness meditation.

**Methods:**

Records were retrieved from PubMed, Web of Science, EBSCO, and Science Direct. A total of 1,612 randomized controlled trial studies on the effect of mindfulness meditation on pregnancy depression were collected. 10 studies with 658 subjects meeting the inclusion criteria were extracted and analyzed by Revman 5.3 to evaluate study qualities. Stata 12.0 was used for the meta-analysis.

**Results:**

Mindfulness meditation had a positive effect on depression during pregnancy [standardized difference of the mean (SMD) = −0.786, 95% confidence interval (−1.289, −0.283), *P* < 0.001]. Subgroup analysis showed that mindfulness-based cognitive therapy (MBCT) had the best intervention effect on depression during pregnancy (SMD = 2.795), the best intervention duration was below 4 weeks (SMD = 1.756), applied from the first to the third stage of pregnancy (SMD = 1.024), the frequency guided by experts was less than six times a week (SMD = 2.055) of <60 min each time (SMD = 1.068), and completing homework by themselves every day for 30 mins (SMD = 1.822) was most significant for the improvement of depression during pregnancy. We found high heterogeneity across studies [q = 97.003, DF = 10, I^2^ = 88.0% (*P* < 0.001), I^2^ > 75%]. This may be caused by variance in measurement tools, among which Beck Depression Inventory-II was a significant source of heterogeneity.

**Conclusion:**

Mindfulness meditation can improve the prevention, remission, and reduction of depression during pregnancy and can be used as an auxiliary measure for the clinical treatment of pregnancy depression.

## Introduction

According to a report by the World Health Organization, depression has become one of the major causes of disability worldwide (Friedrich, 2017). During pregnancy, significant changes in hormone levels occur in pregnant women, affecting the regulation of emotions in the brain through changes in neural transmission. This can produce clinical manifestations such as depression, extreme irritability, moodiness, overeating, or loss of appetite (Llewellyn et al., 1997). With the accelerating pace of life, pressure on women is increasing in work and daily life, increasing the prevalence of depression during pregnancy (Séguin et al., 1995; Bunevicius et al., 2009). Depression can be difficult to diagnose during pregnancy, and untreated pregnancy depression can lead to poor delivery results as well as postpartum depression, and in general reduces the quality of daily life of pregnant women (Brand and Brennan, 2009). It is not conducive to the relationship between pregnant women and their family members and negatively affects the women's as well as fetal behavior and future social and cognitive development (Brand and Brennan, 2009). In addition, depression during pregnancy may reduce the immunity of pregnant women, thereby affecting growth and development of the fetus (Field et al., 2008; Grote et al., 2010). Increasing evidence suggests that depression during pregnancy can have negative short- and long-term effects on pregnant women and new-borns, and that these variables have complex interactions (Aleksandra et al., 2015). Previous studies have found that pregnant women are prone to depressive symptoms both before and after delivery. Approximately 20% of pregnant women suffer from prenatal depression, and 12–16% of pregnant women may suffer from postpartum depression (Leung and Kaplan, 2009). Approximately 4–15% of pregnant women have some depressive symptoms during pregnancy, and the incidence of depressive symptoms during mid-and late pregnancy is especially high (O'Hara et al., 1984; Bennett et al., 2004). Obviously, depression during pregnancy has become a public health problem.

Although antidepressants are effective in the treatment of depression, studies have shown that drug intervention in pregnant women may have side effects on the health of the fetus, including premature delivery, low birth weight, respiratory distress, and hypoglycaemia (Grigoriadis et al., 2014). Women who receive medication and have a history of depression choose to give up medication during pregnancy for fear of problems in fetal development, resulting in a recurrence rate of depression of nearly 70% in pregnant women (Roca et al., 2013). Non-drug interventions are safer and more reliable than drug interventions and should be considered for the clinical treatment of depression during pregnancy (Vieten and Astin, 2008). Pregnant women may be more willing to alleviate depression through non-drug treatment during pregnancy (Dimidjian and Goodman, 2014). Physical exercise has been shown to be an effective non-drug treatment for depression (Kvam et al., 2016). However, for pregnant women with a long pregnancy cycle and inconvenient movement, the form, duration, and intensity of physical exercise available may not effectively alleviate the symptoms of depression. Music therapy has a short-term beneficial effect on patients with depression (Aalbers et al., 2017), but its effect on pregnant women with long-term severe depression is unclear. Transcranial magnetic stimulation is used to stimulate the brain, peripheral nerves, and muscles of patients with major depression to improve their symptoms (Thompson, 2020). Although this technology is considered to be low risk for pregnant women and fetuses, the risk of adverse effects on pregnant women cannot be fully excluded due to the small number of observed cases (Kim et al., 2018).

Compared to the above non-drug treatment methods, no adverse effect of mindfulness meditation on pregnant women or fetuses has been found. Currently, mindfulness meditation practice is used in clinics to assist in the treatment of depression (Miller et al., 1995; Speca et al., 2000; Mason and Hargreaves, 2011; Marchand, 2012). Some studies have defined “mindfulness” as an ability to focus on the present without bias (Brown and Ryan, 2003). Therefore, mindfulness meditation aims to produce a mental state that focuses on the present. After more than ten years of research, different mindfulness meditation intervention methods could demonstrably improve the symptoms of patients with depression, including mindfulness-based decompression therapy, cognitive therapy (Ramel, 2004; Kabat-Zinn, 2011), dialectical behavioral therapy (Cameron, 2015), and acceptance and commitment therapy (Blackledge and Hayes, 2001). For example, mindfulness cognitive therapy (MBCT) can effectively treat the recurrence of depression (Silva, 2003), has become an effective intervention to prevent recurrences of depression (Eisendrath et al., 2014), and may reduce the risk of depression in high-risk pregnant women (Dhillon et al., 2017). Empirical studies indicate that MBCP was effective in reducing perceived stress and the risk of depression during pregnancy (Lönnberg et al., 2019). Mindfulness can help women reduce their symptoms of depression and anxiety (Yang et al., 2019). Mindfulness-based training interventions may reduce stress during pregnancy and improve depression in pregnant women (Matvienko-Sikar et al., 2016). A meta-analysis of 23 RCTs found mindfulness meditation to be a relatively mature and experience-supported, common clinical treatment method. The depressive symptoms of meditators improved compared to a control group (Gotink et al., 2015). Mindfulness meditation intervention is thus well suited as a non-drug treatment for prevention and treatment of mental health problems. It has a positive impact on depression during pregnancy and helps reduce subclinical depressive symptoms (Schreiner and Malcolm, 2008). Although the overall impact of mindfulness meditation on depression during pregnancy has been verified in previous research, a limited number of studies have evaluated how the characteristics of the interventions may affect the efficacy. In light of this, we used a meta-analysis to evaluate mindfulness meditation interventions for pregnant women with depression. We evaluated outcome indicators for the impact of mindfulness meditation on pregnancy depression intervention for the relevant literature with randomized controlled trials.

## Materials and methods

### Literature retrieval strategy

**Figure 1:**
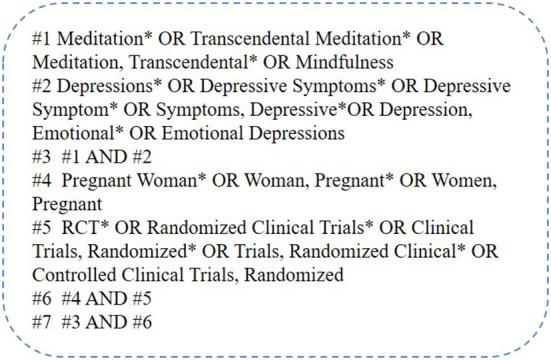
Retrieval strategy.

Records were retrieved from PubMed, Web of Science, EBSCO, and Science Direct. Randomized controlled experimental literature on mindfulness meditation interventions in pregnancy depression was collected by running searches by combining specific search terms such as “Meditation,” “Transcendental Meditation,” “Meditation, Transcendental,” “Mindfulness,” “Depressions,” “Depressive Symptoms,” “Depressive Symptom,” “Symptoms, Depressive,” “Depression, Emotional,” “ Emotional Depressions,” “ Pregnant Woman,” “Woman, Pregnant,” “Women, Pregnant,” “RCT,” “Randomized Clinical Trials.” References cited in the collected results were also searched to supplement the literature. A specific retrieval strategy for Web of Science is shown in Figure 1. The Endnote software was used to manage the references.

### Eligibility criteria

This meta-analysis was carried out following the PRISMA (Preferred Reporting Items For Systematic Reviews and Meta-Analyses) guidelines (Page et al., 2021). The inclusion criteria were designed based on the PICOS (population, intervention, comparison, outcomes, study design) model (Amir-Behghadami and Janati, 2020). Population: pregnant women aged ≥18 years were included, with no limits on pregnancy, nationality, and health status. Intervention: the experimental group received mindfulness meditation intervention, and the control group received no intervention or general nursing measures. Outcomes: depression rating scale scores. Study design: A randomized controlled trial of mindfulness meditation for depression during pregnancy.

We only included reports where full-text information on methods and outcomes was available. Conference summaries, reviews and unfinished research reports were excluded.

### Study selection and data extraction

Two evaluators independently screened the studies. During preliminary screening, irrelevant literature was removed based on the titles and abstracts. After reading the full text, the data to be included in the meta-analysis were extracted and cross-checked. In case of differences of opinion, a third researcher assisted in the judgement. Extracted data include: (I) basic information: first author, literature title, country, publication year; (II) baseline characteristics of subjects: age, population, sample size, pregnancy cycle; (III) specific intervention mode, cycle, frequency, and time; (IV) measurement tools and outcomes.

### Bias assessment

Two reviewers independently evaluated the quality of the literature using Review Manager 5.4.1. Quality was evaluated as “low risk,” “unclear,” or “high risk” depending on the following seven aspects: random sequence generation, allocation concealment, blinding of participants and personnel, blinding of outcome assessment, incomplete outcome data, selective reporting, other bias. In case of differences, a third researcher assisted in the judgement.

### Statistical analysis

Stata 12.0 statistical software was used for the meta-analysis of the included literature. We adopted a random effects model, and used the standardized difference of the mean (SMD) as an index of effect sizes for measurement data. The effect sizes were categorized as small (0.2 ≤ SMD <0.5), medium (0.5 ≤ SMD <0.8), and large (SMD ≥ 0.8). Heterogeneity tests were performed by calculating χ^2^, and the heterogeneity was divided into negligible heterogeneity (I^2^ = 0–24%), medium heterogeneity (I^2^ = 25–50%), large heterogeneity (I^2^ = 50–74%), and non-negligible heterogeneity (I^2^ = 75–100%) (Cohen et al., 1988). If there was statistical heterogeneity, its source was analyzed by conducting subgroup and sensitivity analyses. Egger's tests were used to detect publication bias. The outcome evaluation indices of this study were continuous variables, provided with 95% confidence intervals (CI).

## Results

### Study selection

**Figure 2:**
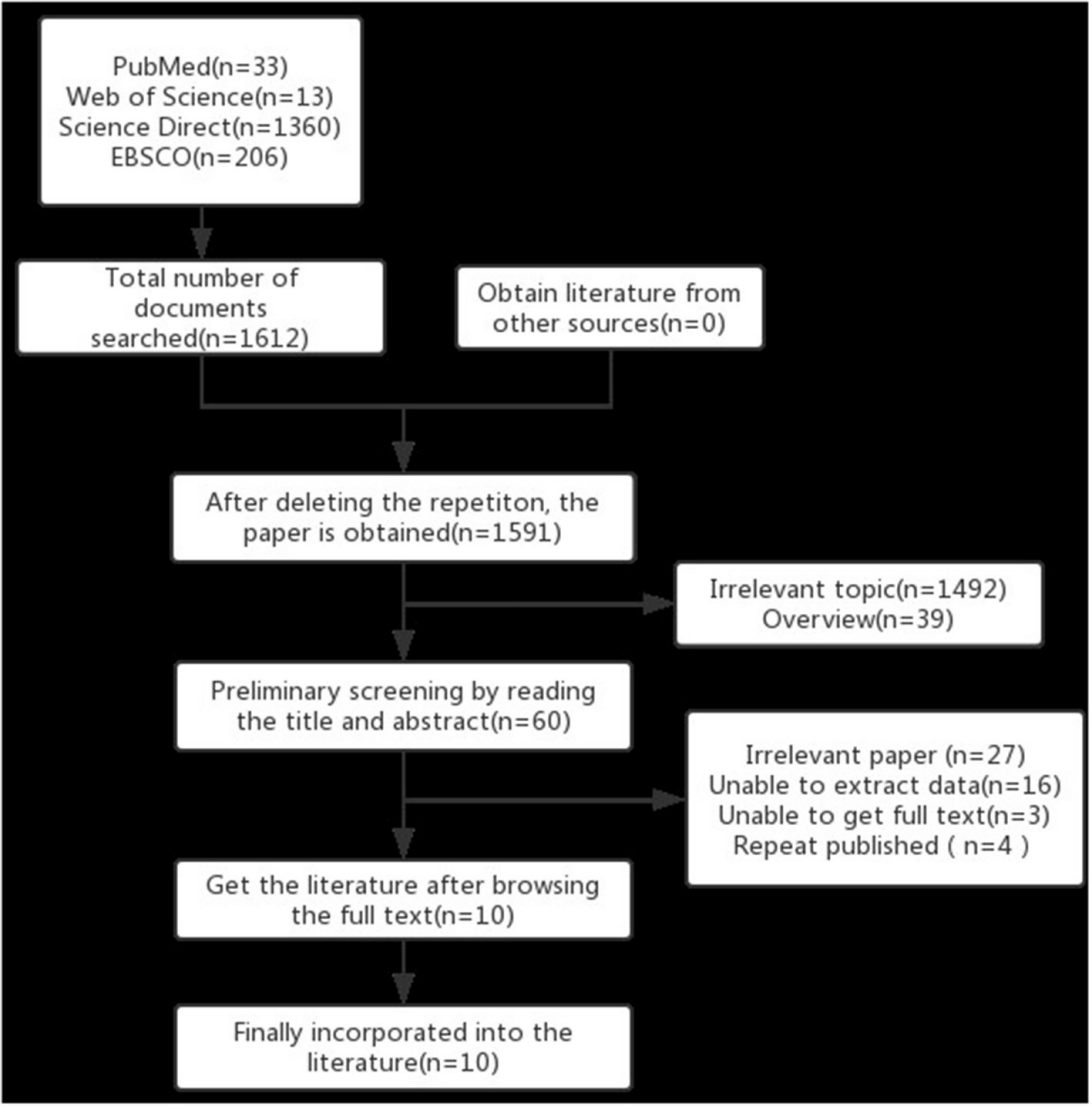
Literature screening process.

A total of 1,612 articles were obtained through database searches and reference collection from relevant papers. After deleting duplicate articles, 1,591 of these were screened. Through reading their titles, abstracts and full text, articles with incomplete, repeated, or inappropriate non-RCT studies were excluded. Finally, ten qualified studies were included. The literature screening process is illustrated in Figure 2.

### Study characteristics and risk of bias

**Figure 3:**
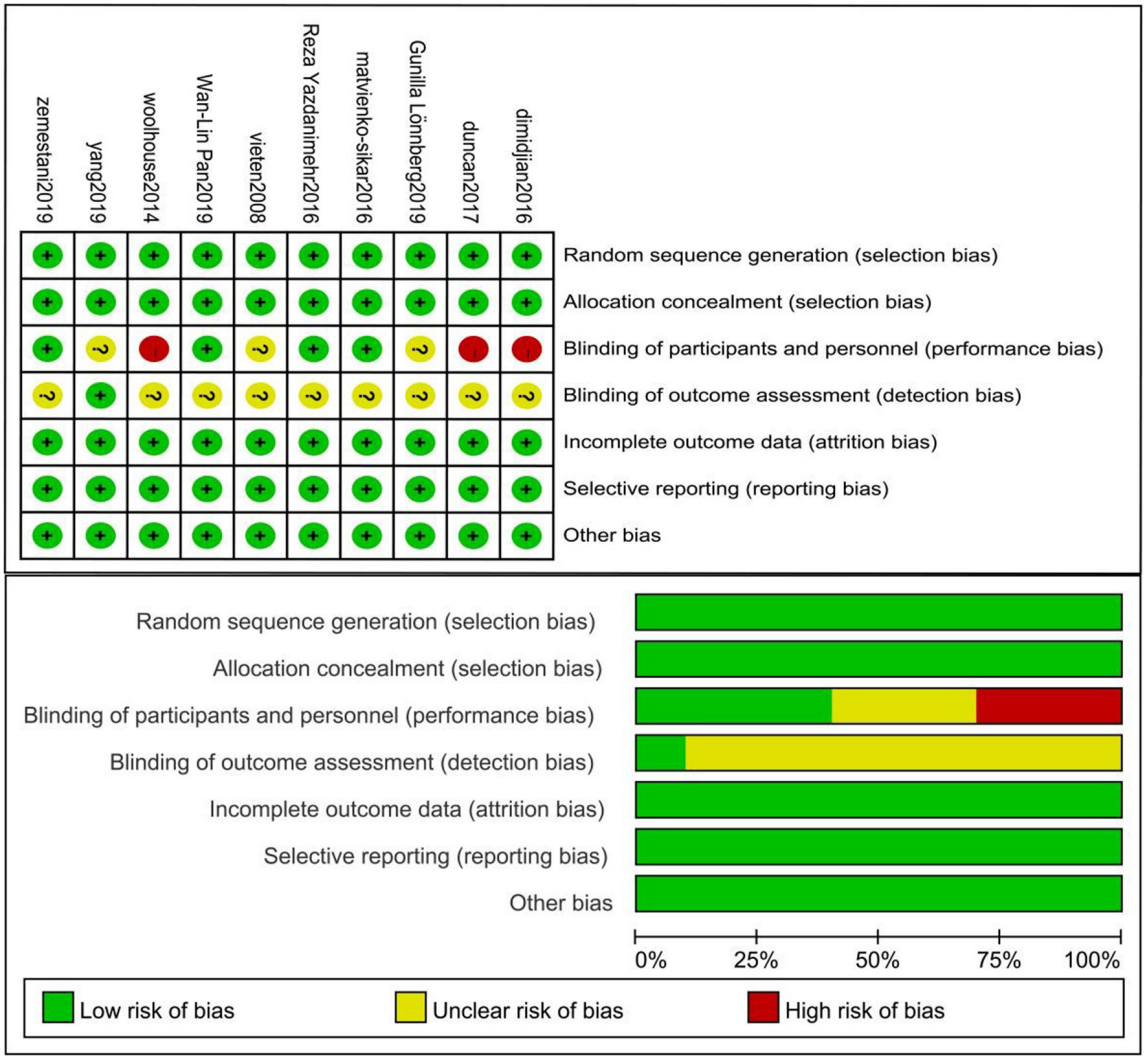
Risk of bias of the included studies.

The basic characteristics of the included studies are presented in Table 1. A total of 685 participants were included in the meta-analysis. A total of 327 and 331 subjects were included in the intervention and control groups, respectively. Among the included studies, one study used a mindfulness-integrated cognitive behavior therapy (MICBT) intervention, two studies used a MBCT intervention, three studies used a mindfulness-based childbirth and parenting (MBCP) intervention, and four studies used a basic mindfulness intervention, including mindfulness practice for 3 days to 8 weeks. The intervention frequency guided by experts was 1–8 times per week, for 6–180 mins each time, and homework time was 0–60 mins every day. Outcome indicators included the following depression scales: (I) The EPDS, a 10-item self-reported questionnaire designed to measure the depressive symptoms of pregnant women during pregnancy and postpartum (Cox et al., 1987); (II) The CES-D Scale, a short self-report scale designed to measure the depressive symptoms of the general population, which is widely used in the epidemiological study of depression (Smarr and Keefer, 2011); (III) The PHQ-9, which is an effective tool for detecting depression, in its Chinese version (Wang et al., 2014); (IV) The DASS, which scores depression, anxiety and stress respectively, and shows good psychometric quality in clinical samples (Parkitny and McAuley, 2010); (V) The BDI-II, a 21 item self-reported questionnaire designed to measure the severity of depressive symptoms (Beck et al., 1996); and VI. The PSS, a 10-item questionnaire designed to assess stress levels in young people and adults (Cohen et al., 1983). The control groups did not receive any meditation intervention. The Cochrane bias risk assessment tool was used to evaluate the quality of the ten included studies. The results of the bias risk assessment are shown in Figure 3.

**Table 1:** Characteristics of the included studies.

**References**	**Study location**	**Sample size**	**Age**	**Participant characteristics**	**Intervention content**	**Outcome instrument**	**Intervention frequency, times per week**	**Intervention period, week**	**Single intervention duration, min**	**Homework per day, min**
Dimidjian et al. (2016)	America	I:24	≥18	Pregnant women with history of depression	MBCT	EPDS	1	8	120	Unknown
		C:31								
Zemestani and Nikoo (2019)	Australia	I:19	≥18	Pregnant women	MBCT	BDI-II	1	3	60	30
		C:19								
Duncan et al. (2017)	America	I:15	Unknown	Third trimester of pregnancy	MBCP	CES-D	2 days and a night	3 days	360	0
		C:13								
Yang et al. (2019)	China	I:62	≥18	24–30 weeks' gestation	Mindfulness	PHQ-9	0.5	8	45–50	20
		C:61								
Yazdanimehr et al. (2016)	Iran	I:30	20–31	Pregnant women who had a gestational age of 1–6 months	MICBT	EPDS	1	8	90	Unknown
		C:33								
Pan et al. (2019)	China	I:39	≥20	Singleton pregnancy between 13 and 28 weeks	MBCP	EPDS	1	8	180	30
		C:35								
Lönnberg et al. (2019)	Sweden	I:75	27–36	First pregnant and gestational 15–22 weeks	MBCP	EPDS	8	8	135	30
		C:89								
Matvienko-Sikar et al. (2016)	Ireland	I:24	27–40	Singleton pregnancy between 10 and 22 weeks	Mindfulness	EPDS	4	3	6	Unknown
		C:12								
Woolhouse et al. (2014)	Australia	I:13	18–50	Between 10 and 34 weeks gestation	Mindfulness	CES-D	1	6	15–20	6–10 min/ 3–4 day
		C:10								
Woolhouse et al. (2014)	Australia	I:13	18–50	Between 10 and 34 weeks gestation	Mindfulness	DASS	1	6	15–20	6–10 min/ 3–4 day
		C:10								
Vieten and Astin (2008)	America	I:13	30–37	Between 12 and 30 weeks gestation	Mindfulness	PSS	1	8	120	60
		C:18								

I, Intervention group; C, Control group.

### Meta-analysis

#### Heterogeneity test

**Figure 4:**
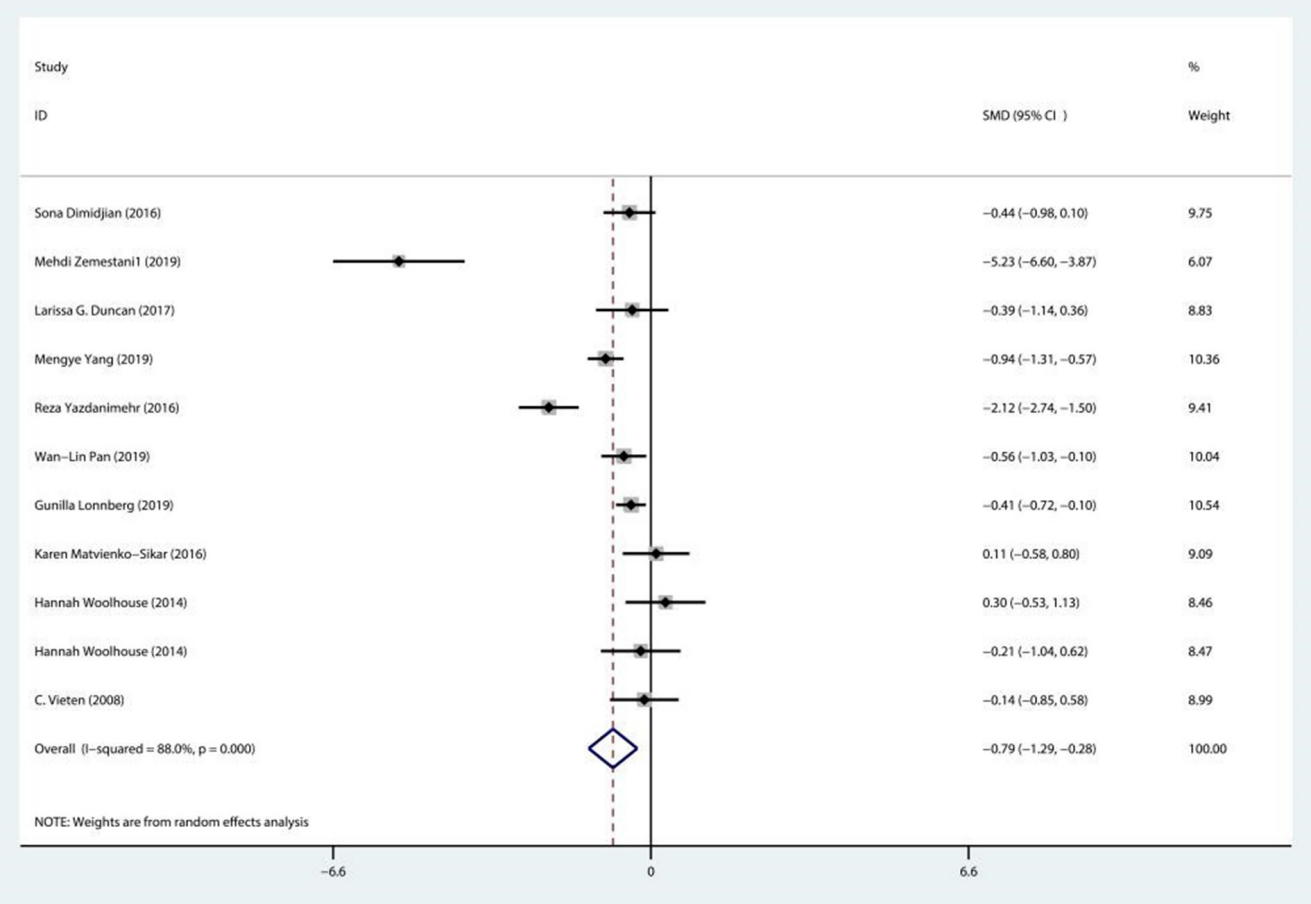
Heterogeneity test of the effect of mindfulness meditation on depression during pregnancy.

The results of the meta-analysis using a random effects model showed that mindfulness meditation had a significant positive effect on depression and significantly reduced depression symptoms during pregnancy [SMD = −0.786, 95% CI (−1.289, −0.283), *P* < 0.001]. Through the heterogeneity test [Q = 97.003, df = 10, I^2^ = 88.0% (*P* < 0.001), I^2^ > 75%], non-negligible heterogeneity was detected; thus, a random effects model was selected for the meta-analysis to explore the source of heterogeneity, as shown in Figure 4.

#### Sensitivity analysis

**Figure 5:**
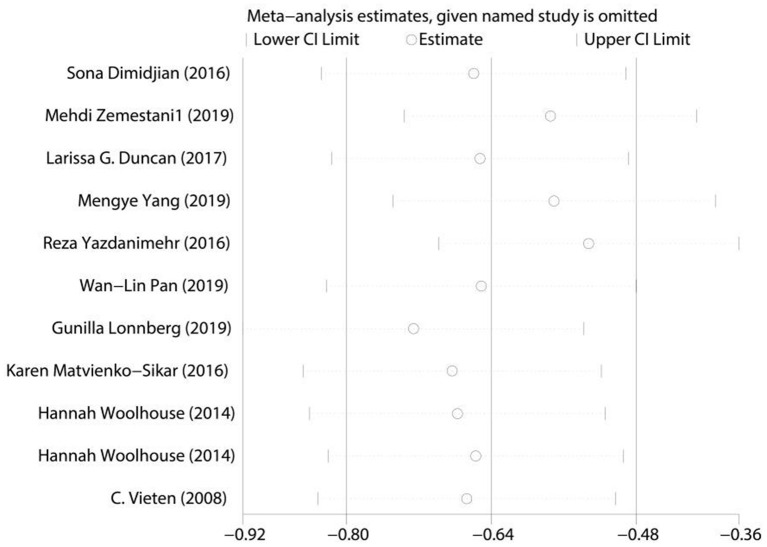
Sensitivity analysis of mindfulness meditation on depression during pregnancy.

We performed sensitivity analysis of the included studies, as shown in Figure 5. Removing the included studies one by one, we estimated interval and plotted the result with 95% CIs in Figure 5. The overall consistency of the included studies is high. Egger's test was used for bias testing and we found no obvious publication bias (*P* > 0.05).

#### Meta regression

To further explore the source of heterogeneity, we conducted univariate meta-regression analysis based on the aspects of country, measurement tool, expert-guided intervention frequency, intervention time, homework time, intervention cycle, the participants' pregnancy cycle, and intervention method. The results of the meta-analysis in Table 2 show that study country, expert guidance intervention frequency, intervention time, homework time, intervention cycle, pregnancy cycle, and intervention methods, had *P*-value above 0.05, indicating that these variables had no significant impact on the heterogeneity among the included studies, while the results show that measurement tool had *P*-value below 0.05.

**Table 2:** Meta-regression analysis of heterogeneity among the effect of mindfulness meditation on depression during pregnancy.

**_ES**	**Coef**.	**Std. err**.	**T**	***P*** **(|t|>T)**	**(95% Conf. interval)**
**Country**					
America	0.0895426	2.132893	0.04	0.968	−5.393235, 5.57232
Australia	−1.198861	2.145898	−0.56	0.600	−6.715067, 4.317345
China	−0.338208	2.256555	−0.15	0.887	−6.138867, 5.462451
Iran	−1.706568	2.618124	−0.65	0.543	−8.43667, 5.023534
Ireland	0.5215077	2.623147	0.20	0.850	−6.221507, 7.264522
_cons	−0.4124042	1.840431	−0.22	0.832	−5.143383, 4.318575
**Outcome**					
BDI-II	−5.019338	1.217595	−4.12	0.006	−7.998687, −2.039989
CES-D	0.1307435	0.8783285	0.15	0.887	−2.018449, 2.279936
EPDS	−0.4673661	0.8252728	−0.57	0.592	2.486736, 1.552004
PHQ-9	−0.727585	1.013035	−0.72	0.500	−3.206393, 1.751223
_cons	−0.2113961	0.7649618	−0.28	0.792	−2.08319, 1.660398
**Guided intervention frequency, times per week**					
<6	−2.038471	1.301185	−1.57	0.156	−5.039008, 0.9620661
>6	−0.641097	1.153203	−0.56	0.593	−3.300387, 2.018193
_cons	0.0460384	1.005095	0.05	0.965	−2.271716, 2.363792
**Length of Each Intervention Session, min**					
60-120	−0.4475529	1.32462	−0.34	0.744	−3.502132, 2.607026
≤ 60	−0.642078	1.192144	−0.54	0.605	−3.391168, 2.107012
_cons	−0.453964	0.9339299	−0.49	0.640	−2.60761, 1.699682
**Daily homework intervention time, min**					
=30	−1.724136	1.717424	−1.00	0.345	−5.684522, 2.236251
<30	−0.4004125	1.585829	−0.25	0.807	−4.05734, 3.256515
_cons	−0.135892	1.484032	−0.09	0.929	−3.558076, 3.286292
**Intervention duration, week**					
<4	−1.711758	1.406292	−1.22	0.258	−4.954673, 1.531156
>6	−0.8135476	1.242693	−0.65	0.531	−3.679203, 2.052107
_cons	0.0460495	1.083053	0.04	0.967	−2.451476, 2.543575
**The subjects' pregnancy period, stages (1:1–14 week,2:15–28 week,3:29 week-born)**					
1–2	−0.471455	2.222238	−0.21	0.839	−5.909075, 4.966165
1–2–3	−0.684741	2.115088	−0.32	0.757	−5.860174, 4.490692
2	−0.0258755	2.703124	−0.01	0.993	−6.640181, 6.58843
2–3	−0.5524524	2.705292	−0.20	0.845	−7.172062, 6.067158
_cons	−0.3865287	1.928135	−0.20	0.848	−5.104506, 4.331449
**Intervention content**					
MBCP	1.664611	1.452565	1.15	0.289	−1.770158, 5.09938
MBCT	−0.420399	1.571095	−0.27	0.797	−4.135448, 3.29465
Mindfulness	1.931379	1.383321	1.40	0.205	−1.339654, 5.202412
_cons	−2.118972	1.260789	−1.68	0.137	−5.100265, 0.8623202

Of the six depression measurement scales used across the studied papers, only the BDI-II showed a significant effect. In a systematic review of the prevalence of depression during pregnancy, the Beck Depression Scale, EPDS, and structured interviews were used to evaluate depression status to evaluate differences between the scales. The results showed that the Beck Depression Scale had more physical items than the Edinburgh Postpartum Depression Scale (Bennett et al., 2004), and the method of self-reported measurement used in the Beck Depression Scale is prone to subjective human factors. This may be the cause of the observed heterogeneity.

#### Subgroup analysis

Subgroup analyses were conducted based on intervention methods, measurement tools, intervention time, intervention cycle, expert guidance intervention frequency, country, pregnancy cycle, and homework time.

The frequency, cycle, and time of mindfulness meditation intervention for regular meditators were as follows: 14% had mindfulness meditation 1–2 times a week, 69% 3–6 times a week, and 17% seven times a week or more. 19% trained for 1–20 mins each time, 72% for 21–45 mins, and 10% for 45 min or more (Lykins and Baer, 2009). The pregnancy cycle was classified into three stages, the first stage from 1 to 14 weeks, the second stage from 15 to 28 weeks, and the third stage from week 29 up to the birth of the baby (Dahlen, 2007).

The results of the subgroup analysis (Table 3) showed that mindfulness cognitive therapy (MBCT) had the best intervention effect on depression during pregnancy (SMD = 2.795), and the outcome was best measured by EPDS. The best intervention cycle of mindfulness meditation was under 4 weeks (SMD = 1.756), the pregnancy cycle of the subject ranged from the first stage to the third stage of pregnancy (SMD = 1.024), the guidance frequency, time of expert intervention, and time of homework were less than six times the expert guidance per week (SMD = 2.055), each time ≤ 60 min (SMD = 1.068), with participants were required to complete a mindfulness meditation intervention of 30 min (SMD = 1.822) as homework every day.

**Table 3:** Subgroup analysis of the effect of mindfulness meditation on depression during pregnancy.

**Group**	**Study**	**Heterogeneity**		**Results of meta-analysis**		
		**I^**2**^**	***P***	**SMD, 95%CI**	**Z**	***P***
Total	11	88.0%	0.000	−0.786(−1.289,−0.283)	3.06	0.002
**Outcome**						
EPDS	5	86.1%	0.000	−0.679(−1.275, −0.082)	2.23	0.026
BDI-II	1	–	–	−5.231(−6.596, −3.865)	7.51	0.000
CES-D	3	0.0%	0.477	−0.099(−0.538, 0.340)	0.44	0.660
PHQ-9	1	–	–	−0.939(−1.312, −0.566)	4.94	0.000
DASS	1	–	–	−0.211(−1.038, 0.615)	0.50	0.616
**Intervention content**						
MBCT	2	97.6%	0.000	−2.795(−7.485, 1.895)	1.17	0.243
MBCP	3	0.0%	0.860	−0.451(−0.695, −0.206)	3.62	0.000
Mindfulness	5	70.0%	0.010	−0.233(−0.769, 0.304)	0.85	0.395
MICBT	1	–	–	−2.119(−2.740, −1.498)	3.06	0.002
**Length of each intervention session, min**						
≤ 60	5	92.9%	0.000	−1.068(−2.273, 0.137)	1.74	0.082
61–120	3	90.9%	0.000	−0.904(−2.092, 0.285)	1.49	0.136
>120	3	0.0%	0.860	−0.451(−0.695, −0.206)	3.62	0.000
**Intervention duration, week**						
<4	3	95.8%	0.004	−1.756 (−4.242, 0.730)	1.38	0.166
4-6	2	0.0%	0.000	0.045(−0.540, 0.631)	0.15	0.879
>6	6	82.5%	0.000	−0.760(−1.218, −0.301)	3.25	0.001
**Guided Intervention Frequency, times per week**						
<6	3	94.9%	0.000	−2.055(−3.960, −0.149)	2.11	0.035
=6	2	0.0%	0.389	0.045 (−0.540, 0.631)	0.15	0.879
>6	6	83.4%	0.000	−0.597(−1.122, −0.072)	2.23	0.026
**Country**						
America	3	0.0%	0.790	−0.346(−0.719, 0.028)	1.81	0.070
Australia	3	95.9%	0.000	−1.654(−4.432, 1.123)	1.17	0.243
China	2	35.2%	0.214	−0.777(−1.143, −0.410)	4.15	0.000
Iran	1	–	–	−2.119(−2.740, −1.498)	6.69	0.000
Sweden	1	–	–	−0.412(−0.723, −0.102)	2.60	0.009
Ireland	1	–	–	0.109(−0.584, 0.803)	0.31	0.758
**The subjects' pregnancy stages (1:1–14 week,2:15–28 week,3:29 week-born)**						
1–2–3	5	92.2%	0.000	−1.024(−2.286, 0.238)	1.89	0.112
3	1	–	–	−0.387(−1.137, −0.364)	1.01	0.312
2–3	1	–	–	−0.939(−1.312, −0.566)	4.94	0.000
1–2	3	92.0%	0.000	−0.860(−2.057, 0.337)	1.41	0.159
2	1	–	–	−0.412(−0.723, −0.102)	2.60	0.009
**Daily homework intervention time, min**						
<30	7	83.0%	0.000	−0.559(−1.131, 0.013)	1.91	0.056
=30	3	95.6%	0.000	−1.822(−3.315, −0.329)	2.39	0.017
>30	1	–	–	−0.136(−0.850, 0.578)	0.37	0.709

## Discussion

In the ten studies included here, the experimental group showed improved symptoms of depression during pregnancy compared to the control group, indicating that mindfulness meditation can effectively improve negative emotions and can prevent, control, and reduce the incidence of depression during pregnancy. For example, in trials of mindfulness meditation intervention for pregnant women, the results showed that mindfulness meditation was a feasible treatment for the intervention of depression (Pan et al., 2019; Kubo et al., 2021). In trials of MBCT intervention on perinatal women with depressive disorder, MBCT was effective for treatment (Miklowitz et al., 2015; Krusche et al., 2018). In a trial of pregnant women with a history of depression, the recurrence of depression and the severity of depressive symptoms in the MBCT-PD experimental group were significantly improved compared with the control group, and MBCT-PD was found to be a clinically acceptable scheme and helpful for reducing the risk of depression during pregnancy (Dimidjian et al., 2016).

Through a meta-analysis, this paper discussed the positive effect of mindfulness meditation on pregnancy depression from the perspective of evidence-based medicine. Previous studies have used electroencephalography technology to measure members of the meditation and control groups, and it was found that activation of left prefrontal cortex was significantly enhanced in meditation practitioners, and that the magnitude of the enhancement could predict the magnitude of the increase in antibody concentration, indicating that mindfulness meditation training can improve relevant neurobiological indexes and promote the improvement of immunity (Davidson et al., 2003). Subsequent studies have shown that mindfulness meditation training can increase sensitivity to sensory stimuli and improve responses (Lazar et al., 2011).

According to existing research, mindfulness meditation can improve depressive symptoms through both physiological and psychological mechanisms: (I) A reduction in the level of cortisol, the main hormone of stress, reduces adverse psychological symptoms and negative emotional reactions, increases subjective wellbeing, improves behavioral regulation, and changes brain activity accordingly, indicating that mindfulness meditation has a direct positive effect on the brain (Keng et al., 2011; Crawford et al., 2013). (II) A reduction in blood pressure improves cardiovascular, cerebrovascular, nervous system, and immune function, improves memory and cognition, and may affect gene phenotype (Krygier et al., 2013; Rosenkranz et al., 2013). (III) Adjustment of brain circuits enhances function in the insula, to drive emotional state in a more positive and optimistic direction (Paulson et al., 2013).

This study had some limitations. First, since only published literature was included in this meta-analysis, the comprehensiveness of the research may be affected. Second, a few of the subgroups have a small sample size, for example the subgroup undergoing MICBT, which may affect the accuracy and feasibility of the subgroup analysis. Third, some characteristics of the participants were not sufficiently clear, such as ethnic and regional differences, age, and disease status.

In summary, mindfulness meditation can reduce behavioral impulses of pregnant women by changing cognition and reducing stress, and improving their understanding of their current experience, so as to promote pregnant women to adapt to their new life and avoid negative emotions as much as possible. Based on the above analysis, specific training strategies have been proposed to improve the effectiveness of mindfulness meditation. Female emotions easily change during and after pregnancy, which is related to many factors such as physiology, psychology, and family. Therefore, it is recommended that pregnant women with depressive symptoms undergo mindfulness meditation training for 4 weeks at any time from the first week of pregnancy to before delivery, especially in the second and third stages of pregnancy (i.e., from 15 weeks to fetal birth). The number of expert-guided interventions could be less than six times a week, each time ≤60 min. Moreover, daily self-meditation of 30 min according to the guidance of experts is also recommended. Future research should supplement this information and provide more targeted suggestions for pregnant women with different statuses.

## Data availability statement

The original contributions presented in the study are included in the article/supplementary material, further inquiries can be directed to the corresponding author.

## Author contributions

YL, JC, and BC formed the conception of the study and drafted the manuscript. TW, ZW, and XH collected and analyzed the data. SL contributed to the conception and revision of the study. All authors contributed to the article and approved the submitted version.

## Funding

This paper is supported by the National Social Science Fund Projects The research on intelligent elderly care service mode of sports and medicine integration (No. 21XTY006) and Beijing Social Science Fund Major Projects Theoretical and practical research on the deep integration of national fitness and national health in the new era (No. 20ZDA19).

## Conflict of interest

The authors declare that the research was conducted in the absence of any commercial or financial relationships that could be construed as a potential conflict of interest.

## Publisher's note

All claims expressed in this article are solely those of the authors and do not necessarily represent those of their affiliated organizations, or those of the publisher, the editors and the reviewers. Any product that may be evaluated in this article, or claim that may be made by its manufacturer, is not guaranteed or endorsed by the publisher.

## References

[B1] Aalbers S. Fusar-Poli L. Freeman R. E. Spreen M. Ket J. C. Vink A. C. . (2017). Music therapy for depression. Cochrane Database Syst. Rev. 11, CD004517. 10.1002/14651858.CD004517.pub3 29144545 PMC6486188

[B2] Aleksandra A. Bogossian F. Wittkowski A. (2015). The experience of psychological distress, depression, and anxiety during pregnancy: a meta-synthesis of qualitative research. Midwifery 31, 563–573. 10.1016/j.midw.2015.03.015 25912511

[B3] Amir-Behghadami M. Janati A. (2020). Population, intervention, comparison, outcomes and study (PICOS) design as a framework to formulate eligibility criteria in systematic reviews. Emerg. Med. J. 37, 387. 10.1136/emermed-2020-209567 32253195

[B4] Beck A. T. Steer R. A. Brown G. K. (1996). Manual of Beck Depression Inventory-II. Washington DC: American University.

[B5] Bennett H. A. Einarson A. Taddio A. Koren G. Einarson T. R. (2004). Prevalence of depression during pregnancy: systematic review. Obstet. Gynecol. 103, 698–697. 10.1097/01.AOG.0000116689.75396.5f 15051562

[B6] Blackledge J. T. Hayes S. C. (2001). Emotion regulation in acceptance and commitment therapy. J. Clin. Psychol. 57, 243. 10.1002/1097-4679(200102)57:2<243::AID-JCLP9>3.0.CO 11180150

[B7] Brand S. R. Brennan P. A. (2009). Impact of antenatal and postpartum maternal mental illness: how are the children? Clin. Obstet. Gynecol. 52, 441–455. 10.1097/GRF.0b013e3181b52930 19661760

[B8] Brown K. W. Ryan R. M. (2003). The benefits of being present: mindfulness and its role in psychological well-being. J. Pers. Soc. Psychol. 84, 822–848. 10.1037/0022-3514.84.4.822 12703651

[B9] Bunevicius R. Kusminskas L. Bunevicius A. Nadisauskiene R. J. Jureniene K. Pop V. (2009). Psychosocial risk factors for depression during pregnancy. Acta Obstet. Gynecol. Scand. 88, 599–605. 10.1080/00016340902846049 19308810

[B10] Cameron A. Y. (2015). Dialectical Behavior Therapy (DBT). The Encyclopedia of Clinical Psychology. 10.1002/9781118625392.wbecp531

[B11] Cohen J. Cohen J. Cohen J. W. Cohen J. Cohen J. Cohen J. . (1988). Statistical power analysis for the behavioral science. Technometrics 31, 499–500.

[B12] Cohen S. Kamarck T. Mermelstein R. (1983). A global measure of perceived stress. J. Health Soc. Behav. 24, 385–396. 10.2307/2136404 6668417

[B13] Cox J. L. Holden J. M. Sagovsky R. (1987). Detection of postnatal depression. Development of the 10-item Edinburgh Postnatal Depression Scale. Br. J. Psychiatry 150, 782-786. 10.1192/bjp.150.6.782 3651732

[B14] Crawford C. Wallerstedt D. B. Khorsan R. Clausen S. S. Walter J. A. G. (2013). A systematic review of biopsychosocial training programs for the self-management of emotional stress: potential applications for the military. Evid. Based Complement. Alternat. Med. 2013, 747694. 10.1155/2013/747694 24174982 PMC3794660

[B15] Dahlen H. (2007). The 3 Stages of Pregnancy. Sydney, NSW: Australian Parents.

[B16] Davidson R. J. Kabat-Zinn J. Schumacher J. Rosenkranz M. Muller D. Santorelli S. F. . (2003). Alterations in brain and immune function produced by mindfulness meditation. Psychosom. Med. 65, 564–570. 10.1097/01.PSY.0000077505.67574.E3 12883106

[B17] Dhillon A. Sparkes E. Duarte R. V. (2017). Mindfulness-based interventions during pregnancy: a systematic review and meta-analysis. Springer Open Choice 8, 1421–1437. 10.1007/s12671-017-0726-x 29201244 PMC5693962

[B18] Dimidjian S. Felder J. N. Brown A. P. Goodman S. H. Gallop R. Beck A. (2016). Staying well during pregnancy and the postpartum: a pilot randomized trial of mindfulness-based cognitive therapy for the prevention of depressive relapse/recurrence. J. Consult. Clin. Psychol. 84, 134–145. 10.1037/ccp0000068 26654212 PMC5718345

[B19] Dimidjian S. Goodman S. H. (2014). Preferences and attitudes toward approaches to depression relapse/recurrence prevention among pregnant women. Behav. Res. Ther. 54, 7–11. 10.1016/j.brat.2013.11.008 24440577

[B20] Duncan L. G. Cohn M. A. Chao M. T. Cook J. G. Riccobono J. Bardacke N. (2017). Benefits of preparing for childbirth with mindfulness training: a randomized controlled trial with active comparison. BMC Preg. Childbirth. 17, 140. 10.1186/s12884-017-1319-3 28499376 PMC5427564

[B21] Eisendrath S. J. Delucchi K. Bitner R. Fenimore P. Smit M. Mclane M. (2014). Mindfulness-based cognitive therapy for treatment-resistant depression: a pilot study. Psychother. Psychosom. 77, 319–320. 10.1159/000142525 18600038

[B22] Field T. Diego M. Hernandez-Reif M. (2008). Prenatal dysthymia versus major depression effects on the neonate. Infant Behav. Dev. 31, 190–193. 10.1016/j.infbeh.2007.10.004 18037494 PMC2315795

[B23] Friedrich M. J. (2017). Depression is the leading cause of disability around the world. JAMA 317, 1517. 10.1001/jama.2017.3826 28418490

[B24] Gotink R. A. Paula C. Busschbach J. J. V. Herbert B. Fricchione G. L. Myriam H. M. G. . (2015). Standardised mindfulness-based interventions in healthcare: an overview of systematic reviews and meta-analyses of RCTs. PLoS ONE 10, e0124344. 10.1371/journal.pone.0124344 25881019 PMC4400080

[B25] Grigoriadis S. Vonderporten E. H. Mamisashvili L. Tomlinson G. Ross L. E. (2014). Prenatal exposure to antidepressants and persistent pulmonary hypertension of the newborn: systematic review and meta-analysis. BMJ 348, f6932. 10.1097/01.ogx.0000450110.64104.c6 24429387 PMC3898424

[B26] Grote N. K. Bridge J. A. Gavin A. R. Melville J. L. Iyengar S. Katon W. J. (2010). A meta-analysis of depression during pregnancy and the risk of preterm birth, low birth weight, and intrauterine growth restriction. Arch. Gen. Psychiatry 67, 1012. 10.1001/archgenpsychiatry.2010.111 20921117 PMC3025772

[B27] Kabat-Zinn J. (2011). Some reflections on the origins of MBSR, skillful means, and the trouble with maps. Contemp. Buddhism 12, 281–306. 10.1080/14639947.2011.564844

[B28] Keng S. L. Smoski M. J. Robins C. J. (2011). Effects of mindfulness on psychological health: a review of empirical studies. Clin. Psychol. Rev. 31, 1041–1056. 10.1016/j.cpr.2011.04.006 21802619 PMC3679190

[B29] Kim D. R. Wang E. Mcgeehan B. Snell J. Epperson C. N. (2018). Randomized controlled trial of transcranial magnetic stimulation in pregnant women with major depressive disorder. Brain Stimul. 12, 96–102. 10.1016/j.brs.2018.09.005 30249416 PMC6301099

[B30] Krusche A. Dymond M. Murphy S. E. Crane C. (2018). Mindfulness for pregnancy: a randomised controlled study of online mindfulness during pregnancy. Midwifery 65, 51–57. 10.1016/j.midw.2018.07.005 30099285

[B31] Krygier J. R. Heathers J. A. J. Shahrestani S. Abbott M. Gross J. J. Kemp A. H. (2013). Mindfulness meditation, well-being, and heart rate variability: a preliminary investigation into the impact of intensive Vipassana meditation. Int. J. Psychophysiol. 89, 305–313. 10.1016/j.ijpsycho.2013.06.017 23797150

[B32] Kubo A. Aghaee S. Kurtovich E. M. Nkemere L. Quesenberry C. P. Jr. . (2021). mHealth mindfulness intervention for women with moderate-to-moderately-severe antenatal depressive symptoms: a pilot study within an integrated health care system. Mindfulness (N Y) 12, 1387–1397. 10.1007/s12671-021-01606-8 33723491 PMC7947160

[B33] Kvam S. Kleppe C. L. Nordhus I. H. Hovland A. (2016). Exercise as a treatment for depression: a meta-analysis. J. Affect. Disord. 202, 67–86. 10.1016/j.jad.2016.03.063 27253219

[B34] Lazar S. W. Kerr C. E. Wasserman R. H. Gray J. R. Fischl B. (2011). Meditation experience is associated with increased cortical thickness. Neuroreport 16, 1893. 10.1097/01.wnr.0000186598.66243.19 16272874 PMC1361002

[B35] Leung B. M. Y. Kaplan B. J. (2009). Perinatal depression: prevalence, risks, and the nutrition link–a review of the literature. J. Am. Diet. Assoc. 109, 1566–1575. 10.1016/j.jada.2009.06.368 19699836

[B36] Llewellyn A. M. Stowe Z. N. Nemeroff C. (1997). Depression during pregnancy and the puerperium. J. Clin. Psychiatry 58 Suppl 15, 26–32. 9427874

[B37] Lönnberg G. Jonas W. Unternaehrer E. Bränström R. Nissen E. Niemi M. (2019). Effects of a mindfulness based childbirth and parenting program on pregnant women's perceived stress and risk of perinatal depression-Results from a randomized controlled trial. J. Affect. Disord. 262, 133–142. 10.1016/j.jad.2019.10.048 31733457

[B38] Lykins E. L. B. Baer R. A. (2009). Psychological functioning in a sample of long-term practitioners of mindfulness meditation. J. Cogn. Psychother. 23. 10.1891/0889-8391.23.3.226

[B39] Marchand W. R. (2012). Mindfulness-based stress reduction, mindfulness-based cognitive therapy, and Zen meditation for depression, anxiety, pain, and psychological distress. J. Psychiatr. Pract. 18, 233. 10.1097/01.pra.0000416014.53215.86 22805898

[B40] Mason O. Hargreaves I. (2011). A qualitative study of mindfulness-based cognitive therapy for depression. British Journal of Medical Psychology 74(2). 10.1348/000711201160911 11802836

[B41] Matvienko-Sikar K. Lee L. Murphy G. Murphy L. (2016). The effects of mindfulness interventions on prenatal well-being: a systematic review. Psychol. Health 2016, 1–20. 10.1080/08870446.2016.1220557 27539908

[B42] Miklowitz D. J. Semple R. J. Hauser M. Elkun D. Weintraub M. J. Dimidjian S. (2015). Mindfulness-based cognitive therapy for perinatal women with depression or bipolar spectrum disorder. Cognit. Ther. Res. 39, 590–600. 10.1007/s10608-015-9681-9 32063660 PMC7021274

[B43] Miller J. J. Fletcher K. Kabat-Zinn J. (1995). Three-year follow-up and clinical implications of a mindfulness meditation-based stress reduction intervention in the treatment of anxiety disorders. Gen. Hosp. Psychiatr. 17, 192. 10.1016/0163-8343(95)00025-M 7649463

[B44] O'Hara M. W. Neunaber D. J. Zekoski E. M. (1984). Prospective study of postpartum depression: prevalence, course, and predictive factors. J. Abnorm. Psychol. 93, 158–171. 10.1037/0021-843X.93.2.158 6725749

[B45] Page M. J. McKenzie J. E. Bossuyt P. M. Boutron I. Hoffmann T. C. Mulrow C. D. . (2021). The PRISMA 2020 statement: an updated guideline for reporting systematic reviews. BMJ 2021, n71. 10.1136/bmj.n71 33782057 PMC8005924

[B46] Pan W. L. Chang C. W. Chen S. M. Gau M. L. (2019). Assessing the effectiveness of mindfulness-based programs on mental health during pregnancy and early motherhood—a randomized control trial. BMC Preg. Childb. 19, 1–18. 10.1186/s12884-019-2503-4 31601170 PMC6785846

[B47] Parkitny L. McAuley J. (2010). The depression anxiety stress scale (DASS). J. Physiother. 56, 204. 10.1016/S1836-9553(10)70030-8 20795931

[B48] Paulson S. Davidson R. Jha A. Kabat-Zinn J. (2013). Becoming conscious: the science of mindfulness. Ann. NY Acad. 1303, 87–104. 10.1111/nyas.12203 24236866

[B49] Ramel. (2004). The effects of mindfulness meditation on cognitive processes and affect in patients with past depression. *Cogn. Ther*. Res. 28, 433–455. 10.1023/B:COTR.0000045557.15923.96

[B50] Roca A. Imaz M. L. Torres A. Plaza A. Subirà S. Valdés M. . (2013). Unplanned pregnancy and discontinuation of SSRIs in pregnant women with previously treated affective disorder. J. Affect. Disord. 150, 807–813. 10.1016/j.jad.2013.02.040 23566335

[B51] Rosenkranz M. A. Davidson R. J. Maccoon D. G. Sheridan J. F. Kalin N. H. Lutz A. (2013). A comparison of mindfulness-based stress reduction and an active control in modulation of neurogenic inflammation. Brain Behav. Immun. 27, 174–184. 10.1016/j.bbi.2012.10.013 23092711 PMC3518553

[B52] Schreiner I. Malcolm J. P. (2008). The benefits of mindfulness meditation: changes in emotional states of depression, anxiety, and stress. Behav. Change 25, 156–168. 10.1375/bech.25.3.156

[B53] Séguin L. Potvin L. Stdenis M. Loiselle J. (1995). Chronic stressors, social support, and depression during pregnancy. Obstet. Gynecol. 85, 583. 10.1016/0029-7844(94)00449-N 7898838

[B54] Silva P. D. (2003). Mindfulness-based cognitive therapy for depression: a new approach to preventing relapse. Behav. Res. Ther. 41, 629–630. 10.1016/S0005-7967(02)00146-822475168

[B55] Smarr K. L. Keefer A. L. (2011). Measures of depression and depressive symptoms: Beck Depression Inventory-II (BDI-II), Center for Epidemiologic Studies Depression Scale (CES-D), Geriatric Depression Scale (GDS), Hospital Anxiety and Depression Scale (HADS), and Patient Health Questionnaire-9 (PHQ-9). Arthritis Care Res. (Hoboken) 63 Suppl 11, S454–466. 10.1002/acr.20556 22588766

[B56] Speca M. Carlson L. Goodey E. Angen M. (2000). A randomized wait-list controlled trial: the effect of a mindfulness meditation-based stress reduction program on mood and symptoms of stress in cancer outpatients. Psychosom. Med. 62, 613–622. 10.1097/00006842-200009000-00004 11020090

[B57] Thompson L. (2020). Treating major depression and comorbid disorders with transcranial magnetic stimulation. J. Affect. Disord. 276, 453–460. 10.1016/j.jad.2020.07.025 32871677 PMC7505211

[B58] Vieten C. Astin J. (2008). Effects of a mindfulness-based intervention during pregnancy on prenatal stress and mood: results of a pilot study. Arch. Womens Mental Health 11, 67–74. 10.1007/s00737-008-0214-3 18317710

[B59] Wang W. Bian Q. Zhao Y. Li X. Wang W. Du J. . (2014). Reliability and validity of the Chinese version of the Patient Health Questionnaire (PHQ-9) in the general population. Gen. Hosp. Psychiatry 36, 539–544. 10.1016/j.genhosppsych.2014.05.021 25023953

[B60] Woolhouse H. Mercuri K. Judd F. Brown S. J. (2014). Antenatal mindfulness intervention to reduce depression, anxiety and stress: a pilot randomised controlled trial of the Mind Baby Body program in an Australian tertiary maternity hospital. BMC Preg. Childbirth. 14, 369. 10.1186/s12884-014-0369-z 25343848 PMC4215015

[B61] Yang M. Jia G. Sun S. Ye C. Zhang R. Yu X. (2019). Effects of an online mindfulness intervention focusing on attention monitoring and acceptance in pregnant women: a randomized controlled trial. J. Midwifery Womens. Health 64, 68–77. 10.1111/jmwh.12944 30695166

[B62] Yazdanimehr R. Omidi A. Akbari H. Sadat Z. (2016). Mindfulness training and quality of life among pregnant women: a randomized clinical trial. Nurs. Midwif. Stud. 6, e32570. 10.5812/nmsjournal.32570 PMC5045953 27752485

[B63] Zemestani M. Nikoo Z. F. (2019). Effectiveness of mindfulness-based cognitive therapy for comorbid depression and anxiety in pregnancy: a randomized controlled trial. Arch Womens Ment Health. 23, 207–214. 10.1007/s00737-019-00962-8 30982086

